# Genome-Wide Scan for Five Brain Oscillatory Phenotypes Identifies a New QTL Associated with Theta EEG Band

**DOI:** 10.3390/brainsci10110870

**Published:** 2020-11-18

**Authors:** Miguel Ângelo Rebelo, Carlos Gómez, Iva Gomes, Jesús Poza, Sandra Martins, Aarón Maturana-Candelas, Saúl J. Ruiz-Gómez, Luis Durães, Patrícia Sousa, Manuel Figueruelo, María Rodríguez, Carmen Pita, Miguel Arenas, Luis Álvarez, Roberto Hornero, Nádia Pinto, Alexandra M. Lopes

**Affiliations:** 1 IPATIMUP—Instituto de Patologia e Imunologia Molecular da Universidade do Porto, 4200-135 Porto, Portugal; info@miguelrebelo.com (M.Â.R.); igomes@ipatimup.pt (I.G.); smartins@ipatimup.pt (S.M.); alopes@ipatimup.pt (A.M.L.); 2 I3S—Instituto de Investigação e Inovação em Saúde, Universidade do Porto, 4200-135 Porto, Portugal; 3 Grupo de Ingeniería Biomédica, Universidad de Valladolid, 47011 Valladolid, Spain; jesus.poza@tel.uva.es (J.P.); aaron.maturana@gib.tel.uva.es (A.M.-C.); saul.ruiz@gib.tel.uva.es (S.J.R.-G.); robhor@tel.uva.es (R.H.); 4 Centro de Investigación Biomédica en Red en Bioingeniería, Biomateriales y Nanomedicina, (CIBER-BBN), 47011 Valladolid, Spain; 5 Instituto de Investigación en Matemáticas (IMUVA), Universidad de Valladolid, 47011 Valladolid, Spain; 6 Associação Portuguesa de Familiares e Amigos de Doentes de Alzheimer, Delegação Norte, 4455-301 Lavra, Portugal; luis.duraes@alzheimerportugal.org (L.D.); patricia.sousa@alzheimerportugal.org (P.S.); 7 Asociación de Familiares y Amigos de Enfermos de Alzheimer y otras demencias de Zamora, 49021 Zamora, Spain; direccion@alzheimerzamora.com (M.F.); psicologia@alzheimerzamora.com (M.R.); carmenpitag@gmail.com (C.P.); 8 Department of Biochemistry, Genetics and Immunology, University of Vigo, 36310 Vigo, Spain; marenas@uvigo.es; 9 TellmeGen, 46010 Valencia, Spain; luis.alvafer@gmail.com; 10 Centro de Matemática da, Universidade do Porto, 4169-007 Porto, Portugal

**Keywords:** electroencephalography rhythms, genome-wide association study, brain QTL, brain electrophysiology, neurological disorders, frequency spectrum relative power, Alzheimer’s disease, endophenotype

## Abstract

Brain waves, measured by electroencephalography (EEG), are a powerful tool in the investigation of neurophysiological traits and a noninvasive and cost-effective alternative in the diagnostic of some neurological diseases. In order to identify novel Quantitative Trait Loci (QTLs) for brain wave relative power (RP), we collected resting state EEG data in five frequency bands (δ, θ, α, β1, and β2) and genome-wide data in a cohort of 105 patients with late onset Alzheimer’s disease (LOAD), 41 individuals with mild cognitive impairment and 45 controls from Iberia, correcting for disease status. One novel association was found with an interesting candidate for a role in brain wave biology, *CLEC16A* (C-type lectin domain family 16), with a variant at this locus passing the adjusted genome-wide significance threshold after Bonferroni correction. This finding reinforces the importance of immune regulation in brain function. Additionally, at a significance cutoff value of 5 × 10^−6^, 18 independent association signals were detected. These signals comprise brain expression Quantitative Loci (eQTLs) in caudate basal ganglia, spinal cord, anterior cingulate cortex and hypothalamus, as well as chromatin interactions in adult and fetal cortex, neural progenitor cells and hippocampus. Moreover, in the set of genes showing signals of association with brain wave RP in our dataset, there is an overrepresentation of loci previously associated with neurological traits and pathologies, evidencing the pleiotropy of the genetic variation modulating brain function.

## 1. Introduction

Neurons, as excitable cells with electrical properties, generate coherent electric and magnetic fields when they are synchronously activated, being akin to current dipoles that may be recorded by electrodes at the scalp [1]. Electroencephalography (EEG) captures the summed electrical activities of neuron populations and EEG signals co-vary strongly with different levels of arousal and consciousness [1]. These signals can be viewed as reflections of outputs from the regulation of synchronization/desynchronization and functional coupling/decoupling in neuron populations with effect on vigilance, motivation and cognitive processes [2,3].

Brain waves recorded as electrophysiological signals harbor an impressive amount of information, thus offering a noninvasive and cost-effective alternative into the investigation of neurophysiological mechanisms. Neural networks change with development, age, disease and personal experience, and abnormalities in the oscillatory activity are associated to neurological disorders such as schizophrenia, autism spectrum disorder (ASD), attention deficit hyperactivity disorder (ADHD) and Alzheimer’s disease (AD) [4,5,6,7,8,9,10]. The mechanisms of synchronization/desynchronization of thalamocortical and ascending activity systems can be analyzed by measuring the amplitude or source activity of resting-state eyes-closed cortical EEG rhythms and can reveal the effects of AD on brain function [4]. In this regard, abnormally reduced spectral coherence in α and β rhythms has been associated with AD, as well as an increase of δ and θ rhythms, contributing with valuable information to build a prediction pipeline for the disease [7,8].

All current evidence suggests that oscillations are essential components in neural computation and not just biomarkers of the process. These manifestations are amongst the most heritable traits in humans [11,12], since individual differences across the EEG frequency spectra in adult population are largely determined by genetic factors. Generally, hereditability is highest around the α frequency band and lower in θ and δ bands [13]. These signals may act as an intermediate phenotype between genetics and behavior, representing endophenotypes [14,15].

The use of brain endophenotypes, such as EEG, has been proposed as a valid approach to uncover the contribution of a given variant to disease, even with modest sample sizes (an important advantage of quantitative phenotypes) [14], by reducing the inherent complexity of behavioral and neurological traits [16,17]. Indeed, although genome-wide association studies (GWAS) have played an important role in uncovering many genetic variants for some conditions, this method has not been so successful for psychiatric and neurological disorders [18]. This is probably due to a more complex genetic architecture of these phenotypes, which reflects the intricacy of the underlying brain processes. Nevertheless, as EEG patterns also likely come from a polygenic model of inheritance, alternative approaches to maximize power to detect relevant biological pathways without large sample sizes are needed. Gene-based and expression-based enrichment analyses increase the power to detect genes affecting the phenotype under study [19,20,21].

Only a few studies to date have searched for genetic variation associated to different brain oscillatory traits, using individuals affected by neurological diseases and controls, and the number of genes identified is still small [22]. We hypothesized that additional quantitative trait loci (QTLs) for brain waves at different frequency bands could be uncovered when using data from cohorts with different EEG phenotypic ranges. For this purpose, resting state EEG data from a novel cohort of 105 patients with late onset Alzheimer’s disease (LOAD), 41 individuals with mild cognitive impairment (MCI) and 45 controls from Iberia were analyzed by means of a genome-wide QTL analysis for brain wave relative power (RP) in five conventional EEG frequency bands (δ, θ, α, β1, and β2), corrected for disease status. With this strategy, we aimed to identify new genes associated with EEG endophenotypes. Our findings contribute a number of interesting candidate genes with a role in brain function.

## 2. Materials and Methods

### 2.1. Subjects

The current study included 105 LOAD patients from North Portugal (*n* = 42) and from the Spanish autonomous community of Castile and León (*n* = 63) with a clinical diagnosis of dementia due to LOAD. AD patients were diagnosed following the criteria of the National Institute on Aging and Alzheimer’s Association (NIA-AA) [23]. The severity of cognitive impairment in AD was assessed by Mini-Mental State Examination (MMSE) test. Patients were classified in the four main stages of AD development: mild (MIL), moderate (MOD), and severe Alzheimer’s disease (SEV).

In addition, 41 MCI subjects and 45 elderly controls with no history of neurological or major psychiatric disorders from both geographical regions were also included in the study. In total, 191 individuals were analyzed. The age of the subjects ranged between 63 and 97 years old, with a mean age of 79 years old for controls, 84 for individuals with MCI and 81 for LOAD patients.

This project has been approved by the Ethics Committee of the University of Porto (CEUP) (report # 38/CEUP/2018), and written informed consents were obtained from all participants, family or legal representatives.

### 2.2. EEG Recording and Processing

Resting state EEG was acquired using a 19-channel EEG system (Nihon Kohden Neurofax JE-921A, Tokyo, Japan) at a sampling frequency of 500 Hz. EEG signals were recorded at electrodes F3, F4, F7, F8, Fp1, Fp2, T3, T4, T5, T6, C3, C4, P3, P4, O1, O2, Fz, Cz and Pz of the International 10–20 System and then re-referenced by means of common average referencing. Subjects were asked to remain awake with eyes closed during EEG acquisition. Each five-minute EEG recording was processed by independent component analysis (ICA), digital filtering using a Hamming window bandpass finite impulse response (FIR) filter in the band of interest (1–30 Hz) and by selecting 5 s artifact-free epochs by visual inspection [24]. Frequency bins were defined as the conventional EEG frequency bands: δ (1–4 Hz), θ (4–8 Hz), α (8–13 Hz), β1 (13–19 Hz) and β2 (19–30 Hz). The metric used to describe the distribution of the spectral content of the signals and explore associations was RP. RP quantifies the relative contribution of each frequency band to the global power spectrum. It is calculated from the power spectral density (PSD) function by summing the contribution of each spectral component in a specific band [24].

### 2.3. Genotyping and Genome-Wide Association Analysis

Saliva was collected from participants either using Oragene DNA (OG-500) self-collection kits (DNA Genotek, Ottawa, Canada) or cotton sterile buccal swabs. DNA was then extracted from the liquid saliva samples using the prepIT DNA extraction kit (DNA Genotek, Ottawa, Canada) and using the Citogene extraction kit (Citomed, Odivelas, Portugal) for the buccal swabs following the manufacturer’s protocols. Samples were genotyped with the Axiom Spain Biobank Array (Thermo Fisher, Waltham, Massachusetts, USA), and the genotyping service was carried out at CEGEN-PRB3-ISCIII, Santigo de Compostela, Spain; supported by grant PT17/0019, of the PE I + D + I. Variant calling and quality control pre-analysis were performed using the Affymetrix Power Tools (APT) and *PLINK* [25]. Individuals with outlying missing genotype or heterozygosity rates (between μ ± 3σ), as well as individuals estimated as related through identical-by-descent measurements and markers with significant deviation to Hardy–Weinberg equilibrium (HWE, α = 10^−7^) were removed prior to the analysis. Through Principal Component Analysis (PCA, implemented in SMARTPCA from EIGENSOFT) individuals with divergent ancestry were identified and removed [26]. The final total genotyping rate was 0.996. Differences between populations from PCA were not statistically significant (*p* = 0.53).

Common variants were selected, considering minimum allele frequencies (MAF) above 5%, which resulted in a set of 388,620 variants. For a nominal significance level α = 0.05, this set of variants leads to a Bonferroni genome-wide significance threshold of 1.29 × 10^−7^, when considering each of the five frequency bands.

To detect association between variants and brain EEG signals, we implemented a multivariable linear regression model in *PLINK*. The regression was done with 5 continuous variables, corresponding to each of the 5 different EEG frequency bands selected for analysis (δ, θ, α, β1, and β2). The model included 5 covariates to correct for residual population substructure: the first 2 principal components (PC), age (since RP suffer important changes across the lifespan [27]), sex and disease status (encoded as 0: control; 1: MCI; 2: MIL; 3: MOD; 4; SEV):

Y = *a* + *b1*.ADD + *b2*.PC1 + *b3*.PC2 + *b4*.AGE + *b5*.SEX + *b6*.dstatus + *ϵ*

being *b1* the coefficient for the applied additive genotypic model of association between SNP and EEG frequency band (ADD). PCs were computed using Eigensoft’s smartpca for the 191 individuals [28]. With this model, all SNP effects found in this study will mostly reflect the genetic effects on brain electrophysiology.

The cutoff value used to evaluate associations was the Bonferroni genome-wide significance threshold of 1.29 × 10^−7^, but a less stringent cutoff of 5 × 10^−6^ was also applied (since our sample size is small) for gene-based analysis.

### 2.4. Common Variant QTL and eQTL Enrichment Analysis

Considering the five linear regressions, top candidate variants were selected considering the significance level α = 0.01 (= 0.05/5). This resulted in the analysis of a set of 16,575 variants.

Functional annotation of GWAS results, comprising gene identification and prioritization by positional, expression-QTL (eQTL) and chromatin interaction (CI) mapping, as well as gene-based pathway and tissue enrichment, was carried out using FUMA (https://fuma.ctglab.nl/) to find likely causal relations from the summary statistics provided (rsID, *p*-value) [21]. SNPs were mapped to genes up to 10 kb apart and the r^2^ threshold of the linkage disequilibrium (LD) to define independent significant SNPs was set to 0.8 (≥). SNPs were filtered based on chromatin state in the brain (accessibility of genomic regions every 200 bp based on 15 categorical states) for state ≤ 7 (open in given tissue/cell type). Gene eQTLs were mapped to Genotype-Tissue Expression (GTEx) V8 Brain tissues filtered by raw *p*-values (α = 5 × 10^−4^) and chromatin state in the brain ≤ 7. Datasets of brain tissues and cell types were selected for 3D CI mapping—the significance of interaction threshold was set to false discovery rate (FDR) ≤ 10^−6^, as suggested by Schmitt and colleagues [29]. The promoter region window was set to 250 bp upstream and 500 bp downstream, to overlap transcription start site (TSS) of genes to significantly interacted regions with risk loci. Overlapping genes were used for gene mapping. Only SNPs overlapped with enhancers and promoters from brain epigenomes were mapped. More detailed information on the analyses incorporated in FUMA is available at https://fuma.ctglab.nl/.

The 50 genes with lower *p*-values were selected and tested for gene-based pathway and brain tissue enrichment, for enrichment in curated gene sets from the HGRI GWAS catalog of reported genes, as well as for evaluating protein interactions using STRING v.11.0 (https://string-db.org/) [30,31].

### 2.5. Statistical Power Computation

The computation of the statistical power to detect an association is of paramount importance. The power to detect a variant-trait association can be quantified through the non-centrality parameter (NPC), which is the expected value of a test statistic to detect association when the null hypothesis is true [32]:

NPC = *n* × *R^2^*/(1 − *R^2^*), with *R^2^* = *r^2^* × *q^2^*

where *n* is the sample size, *q^2^* is the proportion of phenotypic variance explained by a specific causal variant in the population, and *r^2^* is the squared LD correlation between the causal variant and the genotyped one. $R^{2}$ is the proportion of variance explained by the genotyped SNP in the population. If the genotypes at the causal locus are in HWE [33,34], then

*q^2^* = 2 × MAF × (1 − MAF) × *β*^2^

where *β* is the effect size of an allele on the phenotype. This assumes that the analysis for detecting an association is computed by regression of the phenotype on the genotype count (zero, one, or two minor alleles). *R^2^* can then be computed as the ratio between *q^2^* and the phenotype’s total variance [35]:

*R^2^* = *q^2^*/var(Y).

## 3. Results

### 3.1. Linear Regression for 5 Oscillatory Phenotypes Identified One New Association

**Figure 1:**
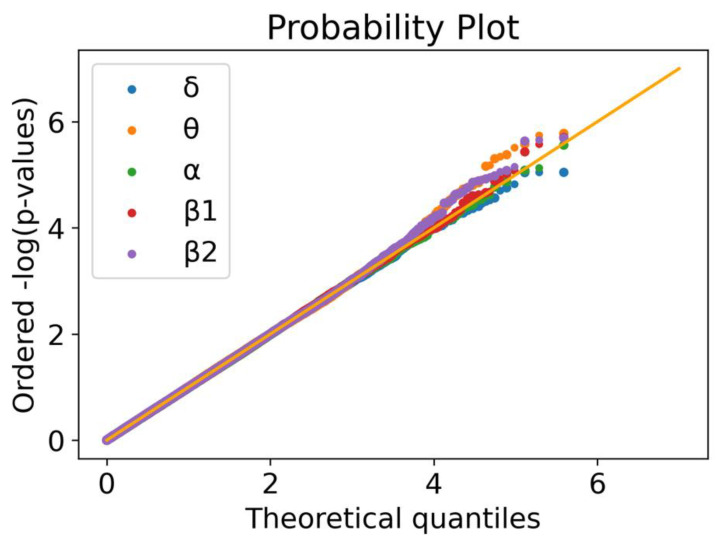
Quantile–quantile plot for the five linear regressions. Ordered quantiles of the log-transformed *p*-values for each of the five linear regressions (δ: blue; θ: yellow; α: green; β1: red; β2: purple) by the theoretical quantiles. A uniform distribution of *p*-values should follow the 45° line in orange.

We tested a total of 388,620 common variants (MAF > 5%). The linear regressions for each of the five frequency bands revealed some deviations from the null distribution (Figure 1). This is more clearly noticed for the θ, β1 and β2 frequency bands. At a significance level *α* = 5 × 10^−6^, 19 highly correlated SNPs were identified, corresponding to 18 independent signals (since two of the loci are in LD), namely 1 in δ, 8 in θ, 2 in α, 4 in β1 and, 4 in β2 (Figure S1A–D, Table 1). With *p*-values ranging from 4.98 × 10^−6^ to 2.64 × 10^−8^, these can be interpreted as strong associations, considering our modest sample size. None of the associated SNPs were detected in more than one phenotype (frequency band) and, to the best of our knowledge, none of these associations were previously reported.

**Figure 2:**
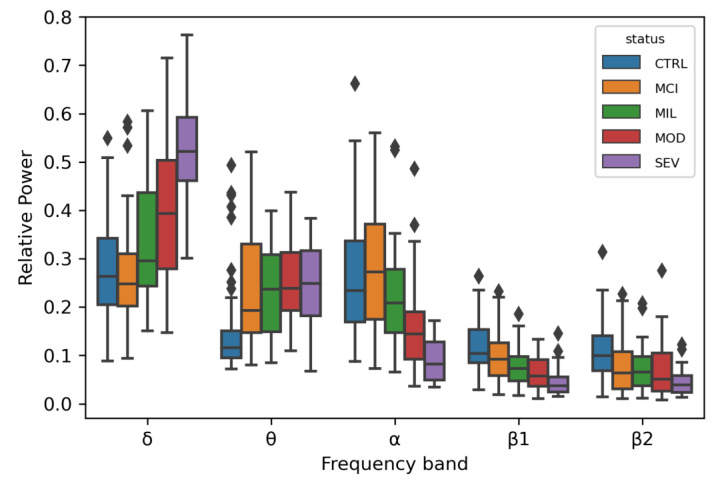
Box plots for the relative power distributions in each frequency band for each disease status. Individuals with outlier relative power values are marked as diamonds. Each color represents a disease status (CTRL: blue; MCI: orange; MIL: green; MOD: red; SEV: purple).

An ANOVA test was computed to evaluate the capacity of the EEG signals for distinguishing between LOAD patients in the different disease stages, subjects with MCI, and controls with normal ageing. RP values distinguished between healthy and affected individuals with statistical confidence, in particular with *p*-values ranging between 1.42 × 10^−7^ and 1.02 × 10^−3^ (Figure 2). This pattern has been observed before in other AD cohorts: an increase in the relative power of slow oscillations (δ and θ rhythms) and a decrease in relative power of fast oscillations (α and β rhythms) [36].

**Figure 3:**
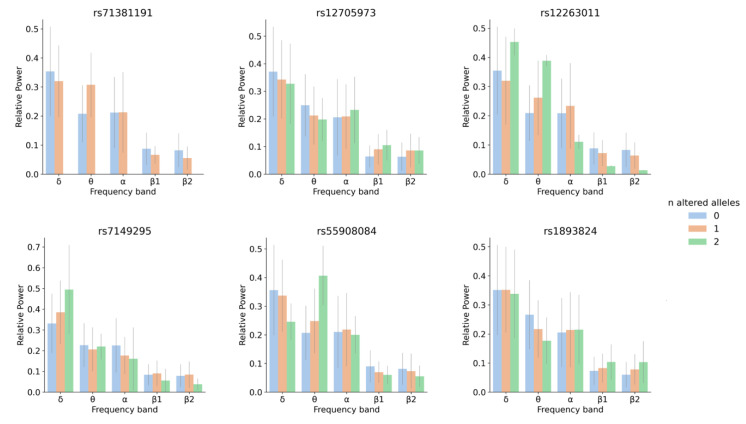
Relative power for each frequency band and genotype for *rs71381191, rs12705973, rs12263011, rs7149295, rs55908084* and *rs1893824.* The number of altered alleles is color coded: blue for individuals with 0 altered alleles (homozygous for the most frequent allele), orange for heterozygous individuals and green for individuals homozygous for the altered (minor) allele.

One variant within a candidate for a role in brain wave biology, *CLEC16A* (C-type lectin domain family 16), passed the genome-wide significance threshold after Bonferroni correction. Individuals with at least one copy of the minor allele of this variant showed higher θ RP values, a tendency also observed in the LOAD patients when compared with controls (Figure 2 and Figure 3). *CLEC16A* is highly expressed in cerebellum (Figure S2) and in cerebellar Purkinje cells in mouse (Allen Mouse Brain Atlas, http://mouse.brain-map.org/gene/show/50215).

### 3.2. SNP-Based Functional Analysis of Most Significant GWAS Hits

For the SNP-based analysis, 16,575 variants from all the five linear regressions were jointly selected (significance level α = 0.01) for functional annotation, gene identification and prioritization by positional, eQTL and CI mapping using FUMA [21]. From this analysis, 15 genomic associated loci corresponding to the lead SNPs that passed the significance thresholds and mapping conditions settings were evaluated, with a total of 18 individually significant SNPs, 3 of them in LD. A total of 30 genes were mapped to these regions (Table 2).

This approach retrieved biologically meaningful results, since all the genomic loci mapped to the most significant SNPs by FUMA, either as eQTL or CI analysis, are brain-related. Brain eQTLs were identified in caudate basal ganglia, spinal cord, anterior cingulate cortex and hypothalamus, while CIs were detected mainly in adult and fetal cortex, neural progenitor cells and hippocampus. Moreover, the genes mapped to the most significant SNPs are highly expressed in various brain tissues (Figure S3).

A few genes particularly relevant for brain function were identified with this analysis. Those more directly linked to the phenotypes under study are highlighted next. *FOXP2* (Forkhead box protein P2) was mapped both by eQTL and CI to *rs12705973* (*p*-value *=* 2.65 × 10^−6^, β1 regression coefficient = 0.02) (Figure S4). This gene encodes a transcriptional repressor that plays a role in synapse formation by regulating SRPX2 levels and was shown to be involved in neural mechanisms mediating the speech development [37,38]. It is highly expressed in the head and tail of nucleus caudatus and putamen, which play a role in movement regulation and other nonmotor actions such as procedural learning, associative learning and inhibitory control of action [39].

Even though the variant *rs12263011* physically maps to an intron of *FRMD4A* (*p*-value *=* 4.98 × 10^−6^, θ regression coefficient = 0.07), by CI it was mapped to *CDNF* (Cerebral dopamine neurotrophic factor) and *SUV39H2* (Histone-lysine N-methyltransferase), in the adult and fetal cortex, respectively (Figure S5). The variant *rs12263011* is embedded in a region with epigenetic promoter marks and thus it may modulate the expression of these genes approximately 0.5 Mb apart, through CI. *FRMD4A* (FERM domain-containing protein 4A) is a scaffolding protein that regulates epithelial polarity and has been previously identified as a genetic risk factor for LOAD and cognitive decline [40,41]. Even though this gene is an interesting candidate for an EEG endophenotype, the mechanism through which *rs12263011* or any of the linked intronic SNPs (Figure S6) may contribute to the phenotype is not clear. There is some evidence supporting this variant may alter a transcription factor binding motif (https://pubs.broadinstitute.org/mammals/haploreg/) and thus have a regulatory effect if it lies within a promoter region with enhancer-like features, regulating distal target genes through chromatin loops [42]. *CDNF* is a trophic factor for dopamine neurons, preventing the 6-hydroxydopamine (6-OHDA)-induced degeneration of dopaminergic neurons in substantia nigra [43]. Neurotrophic factors influence the survival, differentiation and maintenance of neurons in the developing and adult nervous system. *SUV39H2* specifically trimethylates Lys-9 of histone H3, which is a tag for epigenetic transcriptional repression. It is involved in the circadian rhythm by being recruited to the E-box elements of the circadian target genes such as *PER2* or *PER1* [44,45].

FUMA functionally mapped *rs7149295* (an intronic variant within *NIN* gene that encodes the centrosomal protein ninein; *p*-value *=* 4.88 × 10^−6^, δ regression coefficient = 0.09), to *L2HGDH* (L-2-hydroxyglutarate dehydrogenase) by CI in fetal cortex (Figure S7). *L2hgdh* KO mice exhibit white matter abnormalities, extensive gliosis, microglia-mediated neuroinflammation and an expansion of oligodendrocyte progenitor cells (OPCs).

The intergenic variant *rs55908084* (*p*-value *=* 2.65 × 10^−6^, δ regression coefficient = 0.06) was mapped directly to *CACNG4* (voltage-dependent calcium channel gamma-4 subunit), the gene upstream to it. *CACNG4* regulates the activity of L-type calcium channels and the trafficking and gating properties of AMPA-selective glutamate receptors (AMPARs), promoting their targeting to the cell membrane and synapses and modulating their gating properties by slowing their rates of activation, deactivation and desensitization [46].

Finally, *rs1893824* (*p*-value *=* 1.66 × 10^−6^, θ regression coefficient = −0.05 “better”) was identified as an eQTL of the gene in its vicinity, *GALR1* (Galanin receptor type 1), in the anterior cingulate cortex (Figure S8). *GALR1* is a receptor for the hormone galanin with the highest expression level in the adenohypophysis [47], and there is evidence supporting that this receptor modulates impulse control in prefrontal-hippocampal circuitry [48].

### 3.3. Gene-Based Expression and GO Analysis

**Figure 4:**
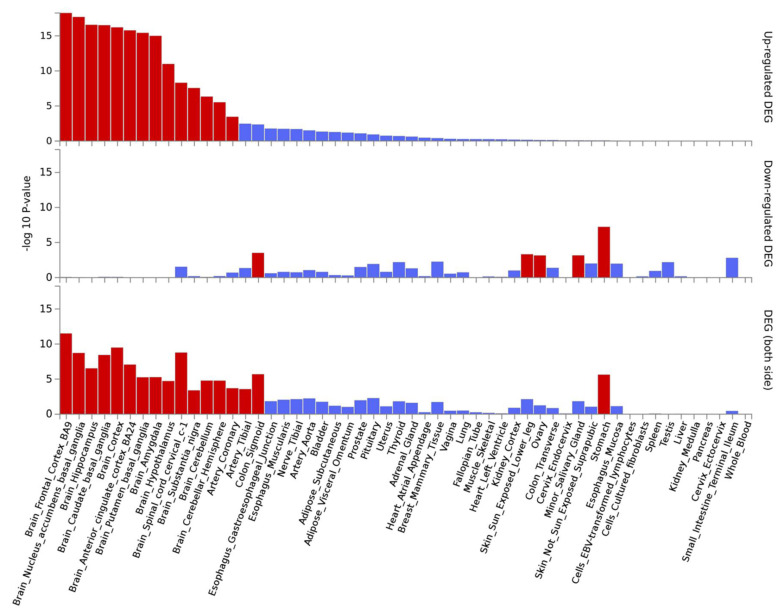
Significantly enriched DEG (differentially expressed genes) for the 50 top genes on GTEx v8 54 tissue types. Log-transformed *p*-values for the enrichment of DEG in each tissue. Significantly enriched tissues are displayed in red (adjusted *p*-value ≤ 0.05), otherwise in blue. The tissues are ordered by their enrichment’s significance on up-regulated genes, and the graph also displays values for down-regulated genes as well as up/down-regulated.

Gene expression analysis for the 15 lead SNPs showed a significant enrichment of genes up-regulated in the hippocampus, anterior cingulate cortex, cortex and caudate basal ganglia (Figure S3). The same analysis was carried out at the gene level, for the 50 most significant genes (Table 3). Each “genescore” computed by FUMA presents a contribution of all variants mapped to it and there is a correction for the gene size. This revealed an enrichment of up-regulated genes in all brain tissues plus coronary tissue (Figure 4).

**Figure 5:**
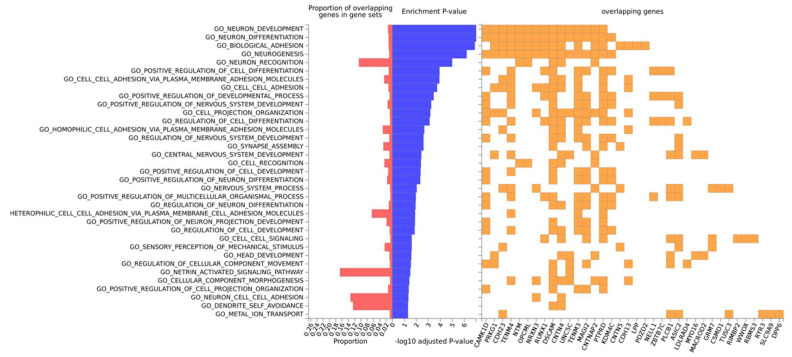
Gene-ontology (GO) most significant categories for biological function. GO biological functions (at the left) are ordered by enrichment log-transformed *p*-value, displayed in blue. Only statistically significant results are shown (*p_adj_* < 0.05). The proportion of overlapping genes (at the bottom) for each GO term (at the left) is displayed in red and for each term the specific genes are identified in yellow.

Gene-ontology (GO) analysis on this set of 50 genes retrieved “Neuron development” and “neuron differentiation” as the topmost enriched categories, and included the following genes: *CAMK1D*, *PRKG1*, *CDH23*, *TENM4*, *NTM*, *OPCML*, *NRXN3*, *RUNX1*, *DSCAM*, *CNTN4*, *UNCSC*, *TENM3*, *MAGI2*, *CNTNAP2*, *PTPRD* and *KDM4C* (neuron differentiation) (Figure 5). All the other significantly enriched categories are related to neuron or head development. By the same token, GO term enrichment for cellular component analysis revealed significantly enriched categories related to neuron parts (Figure S9). As expected, in this set of genes, there is an overrepresentation of associations with brain phenotypes and neurological pathologies such as: chronotype, schizophrenia, Asperger syndrome, brain connectivity, short-term memory, dimensional psychopathology, bipolar disorder and AD (Table 4).

### 3.4. Protein–Protein Interactions

**Figure 6:**
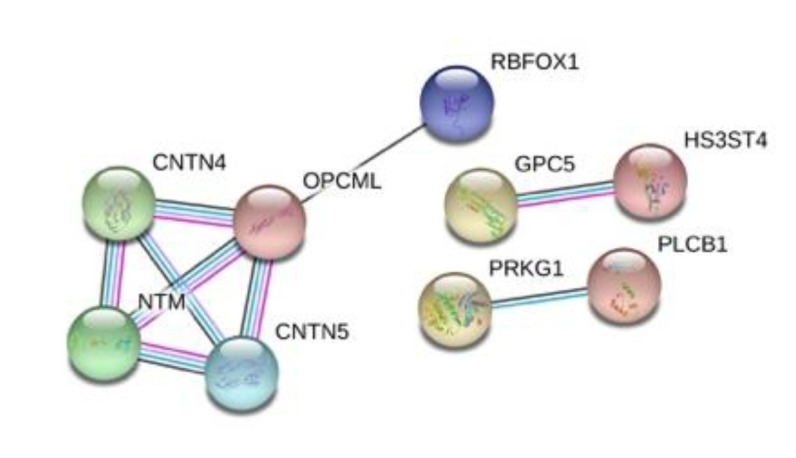
STRING protein interaction analysis for the 50 top protein-coding genes. Inside each node, the 3D structure of each protein is displayed. The edges represent protein–protein interactions. Blue lines represent known interactions from curated databases and pink ones represent experimentally determined interactions. Black lines represent co-expression and purple ones represent homology.

Finally, we performed a protein interaction analysis with STRING for the top 50 protein-coding genes that resulted in a network with significantly more interactions than expected and an enrichment *p*-value = 2.35 × 10^−6^ (Figure 6). Some of the interactions in the network, deserve a closer look. CNTN4 (Contactin-4), mediates cell surface interactions during nervous system development and in conjunction with one of its binding partners, amyloid precursor protein (APP), has been shown to promote target-specific axon arborization, highlighting its importance for the functional development of a behaviorally-relevant parallel visual pathway [49]. CNTN5 (Contactin-5) is a similar molecule but it is exclusively expressed in the central nervous system (CNS), with strong expression in the cortex and hippocampus, and has been associated with ASD [50]. NTM (neurotrimin) is a neural cell adhesion molecule of the immunoglobulin superfamily that appears to regulate the development of neuronal projections and might have a role in mediating estrogen-induced peripheral sympathetic innervation; Ntm-deficient mice have shown a deficit in emotional learning [51,52]. RBFOX1 (RNA binding protein fox-1) regulates alternative splicing in tissue-specific exons, and its cytoplasmic target mRNAs are enriched in genes involved in cortical development and autism [53].

### 3.5. Links to Brain Phenotypes and Neuropathologies

Overall the 16,575 input SNPs were mapped by FUMA to a total of 4078 protein coding genes. In order to identify a possible link between these hits and brain pathophysiology, we inspected the scores for genes previously associated with AD by meta-analysis (Table S1) [54,55]. *CNTNAP2* (Contactin-associated protein-like 2), with 12 mapped SNPs, reveals a strong association (*p*-value *=* 8.92 × 10^−10^). We also inspected genes previously associated with schizophrenia by genome-wide association study and replication (Table S2) [56]. The strongest association was found for *MAGI2* (*p*-value *=* 2.08 × 10^−8^), with 10 mapped SNPs.

The set of the 50 most significant genes in our analysis is also enriched for genes associated with different brain physiological traits, behavioral phenotypes and neurological disorders (Table 3), highlighting the overlap between the biological pathways of brain oscillations and these traits and diseases. The most significant gene in the genescore analysis, *CSMD1* (CUB and sushi domain-containing protein 1), is highly expressed in the CNS, particularly in the frontal cortex (Figure S10) and has been associated with schizophrenia. From the 50 most significant genes, two (*ZBTB7C*, *p*-value *=* 2.29 × 10^−12^; and *FRMD4A*, *p*-value = 9.68 × 10^−11^) were already identified by a single highly significant SNP for each gene. They have also been linked to different brain pathologies. From the 50 most significant genes, *ZBTB7C* (*p*-value = 2.29 × 10^−12^) and *FRMD4A* (*p*-value = 9.68 × 10^−11^) have been previously linked to different brain pathologies [54]. *FRMD4A* has been identified as a genetic risk factor for LOAD and may modulate the disease progression by altering tau [57].

### 3.6. Statistical Power and Effect Size

We evaluated the theoretical statistical power to detect a causal SNP, such as the one identified at genome-wide significance (*rs71381191*), by taking advantage of the relationship between experimental sample size, allele frequency and effect size. The effect sizes for the discovered variants were reported as *b1*—Table 1, referring to the coefficient of the linear regression. Generally, in QTL analyses, where this coefficient is derived from a regression on a continuous variable with widely distributed values, *b1* is not directly interpretable as an effect size per se. However, in our study we considered brain wave RPs, which are normalized measurements, with continuous distribution between 0 and 1. It follows that, in this case, the *b1* coefficient derived from the linear regression is interpretable as the relative effect size. As such, the value of *b1* of 0.11, obtained for the SNP showing statistically significant association, represents an 11% increase in the RP for the θ brain wave, for each additional “effect” allele.

Using the expressions exposed in Section 2.5, with MAF and *β* values for the genotyped genome-wide significant variant (*rs71381191*), we calculated the proportion of phenotypic variance explained by a such causal variant in our population (*q^2^ =* 0.0015) and divided it by the total θ RP variance to get the proportion of phenotypic variance explained by the genotyped SNP (*R^2^ =* 0.14). Finally, the statistical power to detect a SNP explaining 14% of trait variance (such as this one) with a sample size of 191 individuals and assuming our genome-wide significance threshold, was estimated in 62%. We conclude that the relatively large proportion of RP variance explained by *rs71381191* (or a linked variant) has likely contributed to the identification of this significant association in our dataset, for which MAF = 7%.

## 4. Discussion

The easiness and affordability of EEG has made it very appealing as a potential diagnostic tool for several neurological/neurodegenerative diseases. In addition, the characterization of the biological pathways underlying its measurements are thus of high importance. Malone and colleagues, in one of the largest studies involving EEG signals so far, did not find genome-wide significant hits after Bonferroni correction [58]. This is not surprising since the effects of single genetic variants on multifactorial phenotypes such as brain electrophysiological signals are expected to be very small. The largest GWAS of oscillatory power during eyes-closed resting EEG to date found through a gene-based approach that *GABRA2*, a known genetic marker for alcohol abuse disorder and epilepsy, was significantly related to β wave power [22]. Out of twenty-four other genes, three were significantly associated with α power, showing differential expression in two tissues: *GLYCTK* in the hippocampus and *GNL3* and *ITIH4* in the frontal cortex. All of these three genes were previously associated with schizophrenia and bipolar disorder.

### 4.1. Linear Regression for 5 Oscillatory Phenotypes Identified One New θ QTL

We have performed a GWAS and QTL analysis for one brain endophenotype, EEG RP in five frequency bands (δ, θ, α, β1 and β2) in an Iberian cohort of LOAD patients, individuals with MCI and controls, corrected for the disease status, as well as for residual population stratification, age and sex. In spite of the modest sample size, one variant within *CLEC16A*, a C-type lectin, passed the genome-wide significance threshold after Bonferroni correction. *CLEC16A* was shown to participate in the molecular machinery of human leukocyte antigen (HLA) late endosome formation and trafficking, serving as a direct regulator of the HLA-II pathway in antigen-presenting cells [59]. HLA-II is expressed in microglial cells, which are a component of the innate immunity. The relevance of the adaptive immunity in neurodegeneration has been increasingly recognized. An increase in the number of microglial cells, their activation and the disruption of their functions have been demonstrated in neurodegenerative pathologies such as amyotrophic lateral sclerosis (ALS) and frontotemporal dementia [60]. This finding reinforces the importance of immune regulation in brain physiology. Moreover, evidence points to a function of *CLEC16A* in Purkinje cells. This neuronal cell type releases the γ-aminobutyric acid (GABA) neurotransmitter that regulates synaptic plasticity and network oscillations through synaptic inhibition by interneurons that release GABA [61,62]. At the physiological level, cerebellar-evoked prefrontal synchronization in the θ frequency range has been shown to be modulated by GABA, being positively associated with working memory performance [63]. Evidence suggests that the cerebellum likely exerts its control on the cortex by a GABAergic dependent set of interneurons and cerebellar θ-burst stimulation modulates cortical excitability of distant interconnected cortical areas [64]. Recently, the hippocampal expression of a GABA receptor has been associated with β oscillations, supporting the now reported association as being relevant to the electrophysiology of the brain [22]. Considering our present results, another link between GABAergic system and brain electrophysiology surfaces. In addition, the inhibition of CLEC16A protein function also has been shown to lead to motor impairments, Purkinje cell loss and impaired autophagy, pointing to a role in the function and clearance of autolysosomes that culminates in neurodegeneration [65].

### 4.2. SNP and Gene-Based Functional Analysis of Most Significant GWAS Hits and Their Link to Neurological Traits and Pathologies

The analysis with the most nominally significant SNPs from all five linear regressions retrieved a total of 30 candidates, all with functional relevance in brain tissues, as suggested by expression and epigenetic data. Most of the loci physically or functionally mapped by FUMA to the lead SNPs (SNPs that passed the threshold criteria) are involved in brain function, namely neuronal development and maintenance (*CDNF*), cognitive function (*FOXP2*), circadian rhythm (*SUV39H2)* and one is likely a key player in brain electrophysiology (*GALR1*) [39,43,45,66]. Due to their relevance to brain circuitry and physiology these genes have also proven or potential roles in several diseases characterized by impaired cognitive function. Indeed, *FRMD4A* (FERM domain-containing protein 4A) is a scaffolding protein that regulates epithelial polarity and has been previously identified as a genetic risk factor for LOAD [57,67]. This connection to AD is also reinforced by the interaction with PAR3 which regulates CNTNAP2 spatial localization. *CNTNAP2* is required for radial and longitudinal organization of myelinated axons and has been associated with AD and other disorders such as epilepsy, seizures, autism and schizophrenia, and common variants in this gene influence early language acquisition [54,55,68,69,70,71]. Neuropathological analyses in mice with mutations of this gene revealed abnormalities in neuron migration, reduced number of interneurons and abnormal neuronal network activity [72]. Moreover, a homozygous mutation in *FRMD4A* has been linked to a syndrome of congenital microcephaly and intellectual disability [73]. On the other hand, variants in the *ZBTB7C* (Zinc finger and BTB domain-containing protein 7C) have been associated with ischemic injury susceptibility, maybe by modulating the ischemic response via neuronal apoptosis [74]. Another gene among the top hits, *L2HGDH*, is involved in L-2-hydroxyglutaric aciduria (L2HGA), a rare autosomal recessive disorder clinically characterized by a mild psychomotor delay followed by progressive cerebellar ataxia and moderate to severe intellectual disability and a tendency to the development of malignant brain tumors [75]. Moreover, *L2hgdh* deficiency leads to impaired adult hippocampal neurogenesis and late-onset neurodegeneration in mouse brains [76]. It is thus plausible that variants in *L2HGDH* may increase the risk for neurodegenerative disorders. Finally, *GALR1* has been linked to temporal lobe epilepsy (TLE) and galanin agonists inhibit seizures [47].

More indirect evidence for a contribution to neurological diseases exists for other genes in this set. *CDNF*, a trophic factor for dopamine neurons that prevents the 6-hydroxydopamine (6-OHDA)-induced degeneration of dopaminergic neurons in substantia nigra may have a role in the evolution of Parkinson’s disease [43]. *SUV39H,* as an essential part of the circadian system, a timing mechanism responsible for orchestrating many physiological processes including behavior and cognition through epigenetic mechanisms, is a candidate gene for autism susceptibility [77].

As previously mentioned, gene-based approaches increase the power to detect genes affecting the phenotype under study [19,21]. The set of the 50 most significant genes in our analysis is also enriched in genes associated with different brain physiological traits, behavioral phenotypes and neurological disorders, highlighting the overlap between the biological pathways of brain oscillations and these traits and diseases. Indeed, EEG has been a valuable tool in the study of chronotype, brain connectivity, substance abuse, Asperger syndrome, the diagnosis and classification of schizophrenia, and even loneliness [78,79,80,81,82,83].

The most significant gene in the genescore analysis was *CSMD1* (CUB and sushi domain-containing protein 1), which is highly expressed in frontal cortex. Neurophysiological deficits have been observed in *CSMD1* depleted mice, inducing blunted emotional responses, anxiety and depression [84,85], and it has also been linked to schizophrenia. We also inspected genes previously associated with schizophrenia by genome-wide association study and replication [56]. The strongest association was found for *MAGI2* (*p*-value = 2.08 × 10^−8^), with 10 mapped SNPs. This gene encodes a molecule that serves as a scaffold for proteins assembling synaptic protein complexes, therefore, with an essential role in synaptic development and maintenance [86]. In addition, common variants of this gene were associated with cognitive impairment in individuals with schizophrenia, and it has been validated as a strong candidate by genome-wide association [56,87].

### 4.3. Limitations and Future Research Lines

Our study has some limitations that should be addressed. On one hand, the EEG analyses have been performed using the grand-averaged values in order to reduce the dimensionality of the results and simplify statistical analyses. As EEG activity may differ depending on the brain region under study, it could be useful to identify specific affected scalp regions in future studies. For future works, it will be also interesting to acquire and analyze the sleep patterns of the subjects during the night previous to the EEG recording, since some studies reported that disturbances in the sleep-wake cycle and circadian rhythms are common symptoms of AD [88]. On the other hand, it is noteworthy the limited number of subjects analyzed, as already acknowledged, as well as the inclusion of only two closely related populations. The multimodal analysis of larger cohorts, from several diverse populations, will be important to replicate our signals and may uncover other associations between genomic and EEG data.

## 5. Conclusions

One novel association was found with an interesting candidate for a role in brain wave biology, *CLEC16A* (C-type lectin domain family 16), with a variant at this locus passing the adjusted genome-wide significance threshold after Bonferroni correction, reinforcing the importance of the immune regulation in brain function. Moreover, at a significance cutoff value of 5 × 10^−6^, 18 independent association signals were detected. These signals comprise brain expression Quantitative Loci (eQTLs) in caudate basal ganglia, spinal cord, anterior cingulate cortex and hypothalamus, as well as chromatin interactions in adult and fetal cortex, neural progenitor cells and hippocampus. At the same time, in the set of genes showing signals of association with brain wave RP in our dataset, there is an overrepresentation of loci previously associated with neurological traits and pathologies, evidencing the pleiotropy of the genetic variation modulating brain function.

Our results corroborate and strengthen previous findings regarding the biological pathways involved in brain electrophysiology, namely, the role of immunity regulation and GABA neurotransmission, through the identification of a novel candidate gene for brain wave RP modulation. In addition, the complexity inherent to brain phenotypes and the pleiotropy of the variants with potential to modulate brain function is evidenced. In fact, even when dissecting isolated oscillatory endophenotypes, the associated genetic variation has the potential to affect the regulation of other traits and disease risk through diverse mechanisms, such as direct regulation of other genes or by modulating the interaction with other proteins.

## Figures and Tables

**Table 1:** List of significant variants (rsID) at a *p*-value threshold of 5 × 10^−6^. Significant variants (rsID) and their respective *p*-value and regression coefficient (*b1*) for each phenotype (*p*_δ, *p*_θ, *p*_α, *p*_β1 and *p*_β2).

rsID	*p*_δ	*p*_θ	*p*_α	*p*_β1	*p*_β2	*b1*
*rs7516534*			2.76 × 10^−6^			−0.08
*rs12720066*			1.39 × 10^−6^			0.10
*rs12705973*				2.65 × 10^−6^		0.02
*rs10108126*				1.97 × 10^−6^		0.05
*rs10104429*				1.55 × 10^−7^		0.05
*rs7125249*				3.70 × 10^−6^		0.03
*rs6692346*					2.32 × 10^−6^	0.04
*rs4658030*					2.04 × 10^−6^	−0.03
*rs77599684*					9.17 × 10^−7^	0.05
*rs6106856*					2.21 × 10^−6^	0.03
*rs7149295*	4.88 × 10^−6^					0.09
*rs12263011*		4.98 × 10^−6^				0.07
*rs71381191*		2.64 × 10^−8 1^				0.11
*rs12443654*		4.19 × 10^−6^				0.07
*rs9930193*		4.57 × 10^−6^				0.07
*rs55908084*		2.65 × 10^−6^				0.06
*rs9960516 ^2^*		3.09 × 10^−6^				0.05
*rs72919581 ^2^*		1.82 × 10^−6^				0.06
*rs1893824*		1.66 × 10^−6^				−0.05

^1^ Values that passed the Bonferroni threshold of significance. ^2^ SNPs in LD (R^2^ = 0.73).

**Table 2:** Gene mapping of all brain wave associated SNPs with FUMA. Only SNPs showing maximum *p*-value of 0.01 in any of the five linear regressions were selected for this analysis. The table displays the strongly associated genomic loci (Locus), the *p*-value of the lead SNP for each locus (*p*), the individually significant SNPs for each locus (Sig_SNPs), the mapped genes (Gene), the gene product (type) and brain tissues from Genotype-Tissue Expression (GTEx) V8 mapped by gene expression-QTL (eqtlMapts) and by chromatin interaction (ciMapts) to the genomic loci.

Locus	*p*	Sig_SNPs	Gene	Type	eqtlMapts	ciMapts
*1:31318133:C:T*	2.76 × 10^−6^	*rs7516534*	*LAPTM5*	p_coding		Adult_Cortex
			*RN7SKP91*	misc RNA		
			*RP1*-*65J11*.5	antisense		
*1:192427329:C:G*	2.04 × 10^−6^	*rs4658030*				
*7:87169702:A:C*	1.39 × 10^−6^	*rs12720066*				
*7:114313199:A:G*	2.65 × 10^−6^	*rs12705973*	*FOXP2*	p_coding	Brain_Caudate_basal_ganglia	Fetal_Cortex: Neural_Progen_Cell
			*AC073626*.*2*	antisense		Fetal_Cortex: Neural_Progen_Cell
			*MIR3666*	miRNA		
*8:74257947:A:G*	1.55 × 10^−7^	*rs10104429*				
		*rs10108126*				
*10:13865505:A:C*	4.98 × 10^−6^	*rs12263011*	*PRPF18*	p_coding		Fetal_Cortex
			*FRMD4A*	p_coding		
			***CDNF***	p_coding		Adult_Cortex: Fetal_Cortex
			*HSPA14*	p_coding		Adult_Cortex: Fetal_Cortex
			*RP11*-*398C13*.*6*	lincRNA		Fetal_Cortex
			***SUV39H2***	p_coding		Fetal_Cortex
*11:45062339:G:T*	3.70 × 10^−6^	*rs7125249*				
*14:51283148:C:T*	4.88 × 10^−6^	*rs7149295*	***L2HGDH***	p_coding		Fetal_Cortex
			*ATP5S*	p_coding		Fetal_Cortex
			***NIN***	p_coding		Adult_Cortex: Hippocampus
			*RP11*-*286O18*.*1*	antisense		
			*PYGL*	p_coding		Adult_Cortex
*16:11156812:A:G*	2.64 × 10^−8^	*rs71381191*	***CLEC16A***	p_coding		
			*RPL7P46*	pseudogene		
			*RP11*-*66H6*.*3*	antisense		
*16:19375297:C:T*	4.19 × 10^−6^	*rs12443654*	*RPS15A*	p_coding	Brain_Spinal_cord_cervical_c-1	
		*rs9930193*	*CTA*-*363E6*.*2*	lincRNA		
*17:65034162:C:G*	2.65 × 10^−6^	*rs55908084*	***CACNG4***	p_coding		
			*AC005544*.*1*	p_coding		
			*RP11*-*74H8*.*1*	antisense		
*18:21849024:A:G*	9.17 × 10^−7^	*rs77599684*	*OSBPL1A*	p_coding		
			*RN7SL247P*	misc_RNA		
*18:45885064:C:T*	1.82 × 10^−6^	*rs72919581*	*ZBTB7C*	p_coding		
		*rs9960516*				
*18:74959125:G:T*	1.66 × 10^−6^	*rs1893824*	*RP11*-*17M16*.*2*	antisense	Brain_Hypothalamus	
			***GALR1***	p_coding	Brain_Anterior_cingulate_cortex_BA24	
*20:24313473:A:G*	2.21 × 10^−6^	*rs6106856*				

Lead SNPs are underlined, common findings between SNP-based and Gene-based approaches are bolded.

**Table 3:** Genescores for the top 50 most significant genes. The table displays the number of SNPs mapped to each gene (nSNPs) as well as the computed *p*-value for each gene.

Gene	nSNPs	*p*-Value
*CSMD1*	35	3.01 × 10^−16^
*CDH13*	27	4.85 × 10^−14^
*PTPRD*	21	7.89 × 10^−14^
*SORCS2*	21	1.23 × 10^−13^
*RBFOX1*	20	2.05 × 10^−13^
*MACROD2*	20	1.39 × 10^−12^
***ZBTB7C***	12	2.29 × 10^−12^
*RBFOX3*	18	6.64 × 10^−12^
*RUNX1*	14	1.17 × 10^−11^
*WWOX*	23	2.39 × 10^−11^
***FRMD4A***	14	9.68 × 10^−11^
*PLCB1*	13	1.08 × 10^−10^
*LRP1B*	12	1.55 × 10^−10^
*OPCML*	11	2.74 × 10^−10^
*CAMK1D*	13	3.29 × 10^−10^
*FHIT*	13	3.97 × 10^−10^
*PDZD2*	13	4.84 × 10^−10^
*CNTN4*	14	5.03 × 10^−10^
*CDH23*	8	7.60 × 10^−10^
*LPP*	10	8.21 × 10^−10^
*MYO16*	11	8.79 × 10^−10^
*CNTNAP2*	12	8.92 × 10^−10^
*KDM4C*	12	1.06 × 10^−9^
*NTM*	11	1.09 × 10^−9^
*ASIC2*	9	1.13 × 10^−9^
*RYR3*	12	1.22 × 10^−9^
*RPA3-AS1*	13	1.39 × 10^−9^
*OFCC1*	10	1.41 × 10^−9^
*DSCAM*	12	1.46 × 10^−9^
*TMEM132C*	12	1.53 × 10^−9^
*MTUS2*	11	1.98 × 10^−9^
*SLC9A9*	10	2.12 × 10^−9^
*LDLRAD4*	11	3.99 × 10^−9^
*FSTL5*	11	4.06 × 10^−9^
*RBMS3*	10	4.79 × 10^−9^
*TENM4*	13	7.66 × 10^−9^
*NELL1*	10	1.06 × 10^−8^
*TENM3*	8	1.06 × 10^−8^
*PRKG1*	11	1.06 × 10^−8^
*GRM7*	8	1.19 × 10^−8^
*HS3ST4*	8	1.46 × 10^−8^
*CNTN5*	10	1.46 × 10^−8^
*TUSC3*	14	1.60 × 10^−8^
*NRXN3*	7	1.86 × 10^−8^
*DPP6*	11	1.92 × 10^−8^
*UNC5C*	9	2.06 × 10^−8^
*MAGI2*	10	2.08 × 10^−8^
*ADARB2*	8	2.27 × 10^−8^
*RIMBP2*	7	2.53 × 10^−8^
*GPC5*	7	2.59 × 10^−8^

Genes identified by highly significant single SNPs are bolded.

**Table 4:** Enrichment on genesets of reported genes for brain conditions/phenotypes from the NHGRI (National Human Genome Research Institute) GWAS (genome-wide association studies) catalog.

GeneSet	N	*n*	*p*-Value	Adjusted *p*-Value	Genes
Chronotype	556	13	7.05 × 10^−13^	6.39 × 10^−10^	*CNTN5, GPC5, MYO16, NRXN3, RBFOX1, ASIC2, MACROD2, CNTN4, GRM7, FSTL5, MAGI2, CSMD1, PTPRD*
Intracranial aneurysm	72	6	8.85 × 10^−10^	3.97 × 10^−7^	*RBFOX1, DSCAM, RBMS3, FHIT, PDZD2, PTPRD*
Response to amphetamines	33	5	1.09 × 10^−9^	3.97 × 10^−7^	*NELL1, WWOX, CDH13, FHIT, TENM3*
Schizophrenia	827	12	1.43 × 10^−9^	4.31 × 10^−7^	*PRKG1, NELL1, NRXN3, RYR3, CDH13, CNTN4, RBMS3, FHIT, TENM3, MAGI2, CSMD1, KDM4C*
Amyotrophic lateral sclerosis (sporadic)	164	7	3.58 × 10^−9^	8.76 × 10^−7^	*CNTN5, OPCML, RYR3, ASIC2, MACROD2, DSCAM, CSMD1*
Night sleep phenotypes	538	10	3.86 × 10^−9^	8.76 × 10^−7^	*TENM4, CDH13, LRP1B, CNTN4, GRM7, RBMS3, SLC9A9, OFCC1, MAGI2, CSMD1*
Cognitive ability, years of educational attainment or schizophrenia (pleiotropy)	197	6	3.72 × 10^−7^	4.22 × 10^−5^	*CAMK1D, NTM, GPC5, CDH13, LRP1B, CNTN4*
Loneliness (multivariate analysis)	29	3	9.65 × 10^−6^	7.00 × 10^−4^	*PRKG1, CNTN5, PTPRD*
Asperger disorder	6	2	2.97 × 10^−5^	1.54 × 10^−3^	*NTM, FHIT*
Middle childhood and early adolescence aggressive behavior	6	2	2.97 × 10^−5^	1.54 × 10^−3^	*OPCML, CNTN4*
Daytime sleep phenotypes	259	5	3.38 × 10^−5^	1.66 × 10^−3^	*WWOX, CDH13, PLCB1, CNTN4, GRM7*
Brain connectivity	7	2	4.15 × 10^−5^	1.88 × 10^−3^	*MACROD2, CNTN4*
Short-term memory (digit-span task)	7	2	4.15 × 10^−5^	1.88 × 10^−3^	*CDH13, PTPRD*
Dimensional psychopathology (Negative)	9	2	7.10 × 10^−5^	2.53 × 10^−3^	*CDH13, MACROD2*
Hippocampal sclerosis	9	2	7.10 × 10^−5^	2.53 × 10^−3^	*NELL1, SORCS2*
Bipolar disorder (body mass index interaction)	10	2	8.86 × 10^−5^	2.87 × 10^−3^	*CDH23, WWOX*
Aggressiveness in attention deficit hyperactivity disorder	11	2	1.08 × 10^−4^	3.33 × 10^−3^	*NTM, CSMD1*
Alzheimer’s disease (64°)	70	3	1.39 × 10^−4^	3.93 × 10^−3^	*FRMD4A, MYO16, CNTNAP2*

Enrichment on genesets of reported genes for brain conditions/phenotypes (GeneSet) from the NHGRI GWAS catalog [31] for the 50 most significant genes of our study. The table displays the background number of genes in each geneset (N), the sample number of genes present in each geneset (*n*), the respective *p*-value and *adjusted p-*value calculated from the comparison between the background and the sample frequency of genes and the sample genes (Genes).

## Supplementary Materials

The following are available online at https://www.mdpi.com/2076-3425/10/11/870/s1, Figure S1: Linear regression p-values for each frequency band; Figure S2: Gene expression for *CLEC16A* in 54 tissues from GTEx v8; Figure S3: Significantly enriched up-regulated DEGs (differentially expressed genes) for the 15 lead SNPs on GTEx v8 54 tissue types; Figure S4: Circos plot for the *rs12705973* genomic associated locus (*FOXP2*); Figure S5: Circos plot for the *rs12705973* genomic associated locus (*FRMD4A*); Figure S6: Linkage proxies for the variant *rs12263011*, within *FRMD4A*; Figure S7: Circos plot for the *rs7149295* genomic associated locus (*L2HGDH*); Figure S8: Circos plot for the *rs1893824* genomic associated locus (*GALR1*); Figure S9: GO (gene-ontology) most significant categories for cellular component; Figure S10: Gene expression for *CSMD1* in 54 tissues from GTEx v8; Table S1: Genescores of genes previously associated with AD; Table S2: Genescores of genes previously associated with schizophrenia.
