# Supplementary material for: Genome-Wide Scan for Five Brain Oscillatory Phenotypes Identifies a New QTL Associated with Theta EEG Band
Source: Brain Sci. 2020 Nov 18;10(11):870. doi: 10.3390/brainsci10110870 (PMC7698967; doi:10.3390/brainsci10110870)
Supplement: Supplementary file 1 [file brainsci-10-00870-s001.pdf]

brain sciences

Article

Genome-Wide Scan for Five Brain Oscillatory Phenotypes Identifies a New QTL Associated with Theta EEG Band

Miguel Ângelo Rebelo [1,2], Carlos Gómez [3,4,*], Iva Gomes [1,2], Jesús Poza [3,4,5], Sandra Martins [1,2], Aarón Maturana-Candelas [3,4], Saúl J. Ruiz-Gómez [3,4], Luis Durães [6], Patrícia Sousa [6], Manuel Figueruelo [7], María Rodríguez [7], Carmen Pita [7], Miguel Arenas [8], Luis Álvarez [9], Roberto Hornero [3,4,5], Nádia Pinto [1,2,10,*] and Alexandra M. Lopes [1,2]

1 IPATIMUP—Instituto de Patologia e Imunologia Molecular da Universidade do Porto, 4200-135 Porto, Portugal; info@miguelrebelo.com (M.Â.R.); igomes@ipatimup.pt (I.G.); smartins@ipatimup.pt (S.M.); alopes@ipatimup.pt (A.M.L.)
2 I3S—Instituto de Investigação e Inovação em Saúde, Universidade do Porto, 4200-135 Porto, Portugal
3 Grupo de Ingeniería Biomédica, Universidad de Valladolid, 47011 Valladolid, Spain; jesus.poza@tel.uva.es (J.P.); aaron.maturana@gib.tel.uva.es (A.M.-C.); saul.ruiz@gib.tel.uva.es (S.J.R.-G.); robhor@tel.uva.es (R.H.)
4 Centro de Investigación Biomédica en Red en Bioingeniería, Biomateriales y Nanomedicina, (CIBER-BBN), 47011, Valladolid, Spain
5 Instituto de Investigación en Matemáticas (IMUVA), Universidad de Valladolid, 47011 Valladolid, Spain
6 Associação Portuguesa de Familiares e Amigos de Doentes de Alzheimer, Delegação Norte, 4455-301 Lavra, Portugal; luis.duraes@alzheimerportugal.org (L.D.); patricia.sousa@alzheimerportugal.org (P.S.)
7 Asociación de Familiares y Amigos de Enfermos de Alzheimer y otras demencias de Zamora, 49021 Zamora, Spain; direccion@alzheimerzamora.com (M.F.); psicologia@alzheimerzamora.com (M.R.); carmenpitag@gmail.com (C.P.)
8 Department of Biochemistry, Genetics and Immunology, University of Vigo, 36310 Vigo, Spain; marenas@uvigo.es
9 TellmeGen, 46010 Valencia, Spain; luis.alvafer@gmail.com
10 Centro de Matemática da, Universidade do Porto, 4169-007 Porto, Portugal
* Correspondence: npinto@ipatimup.pt (N.P.); carlos.gomez@tel.uva.es (C.G.)

Received: 15 October 2020; Accepted: 10 November 2020; Published: 18 November 2020

Supplementary Materials: The following are available online at www.mdpi.com/xxx/s1, Figure S1: title, Table S1: title, Video S1: title.

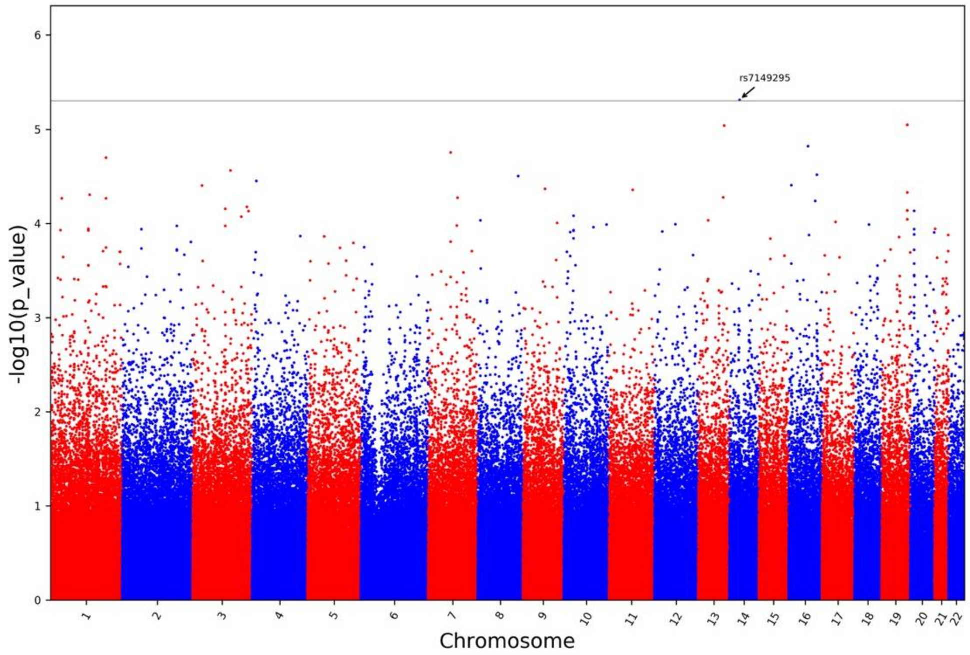

(**a**)

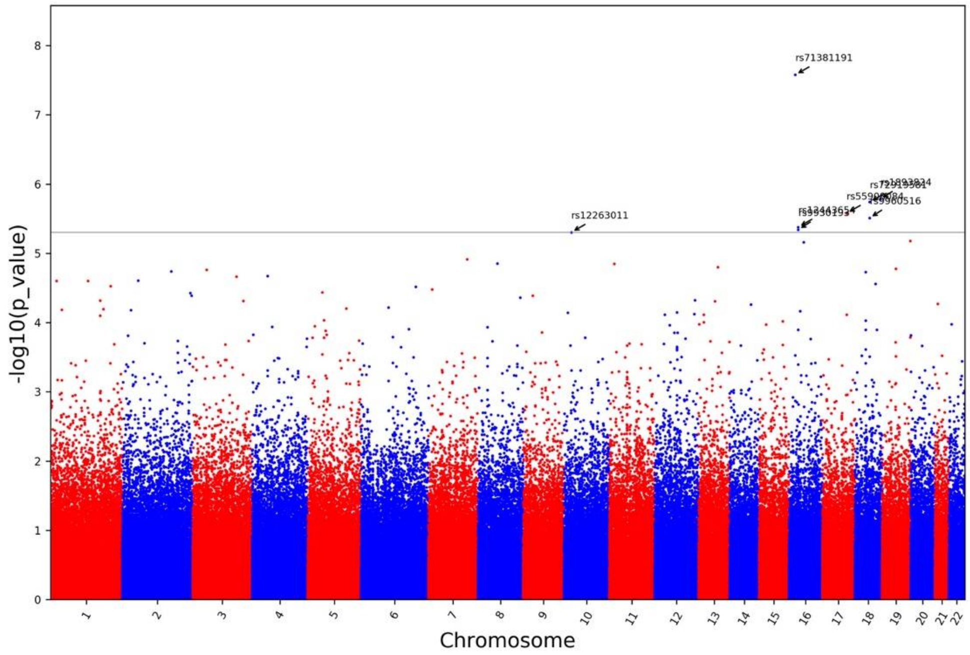

(**b**)

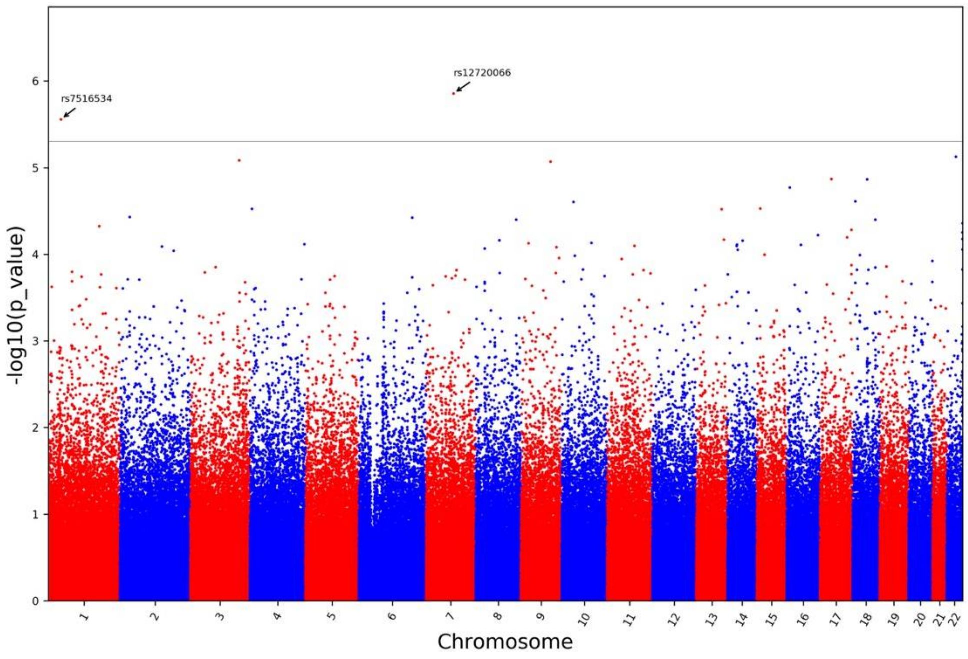
rs12720066
rs7516534
-log10(p_value)
Chromosome

(**c**)

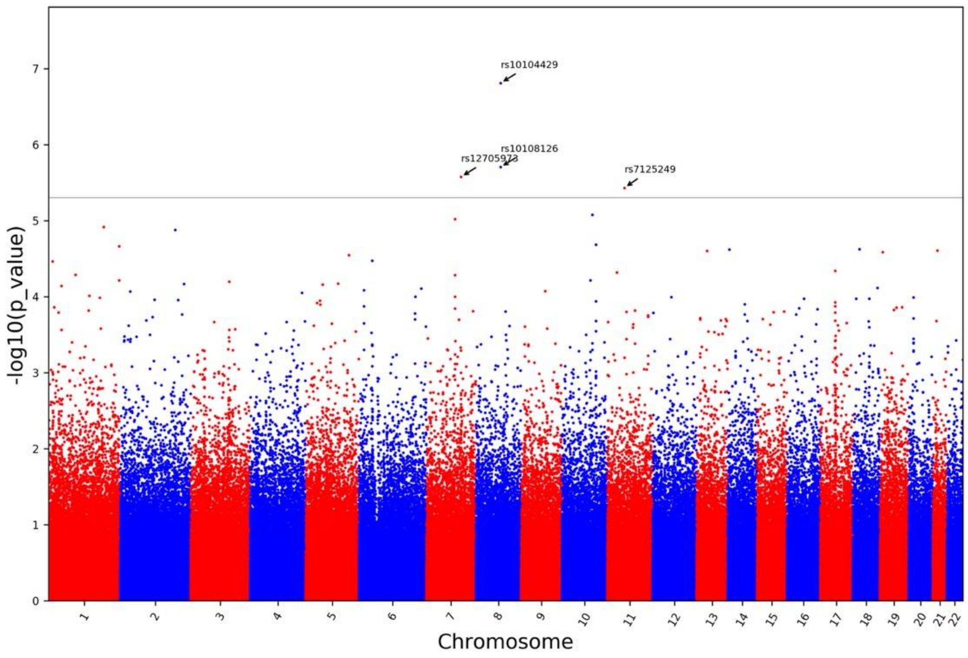
rs10104429
rs10108126
rs12705973
rs7125249
-log10(p_value)
Chromosome

(**d**)

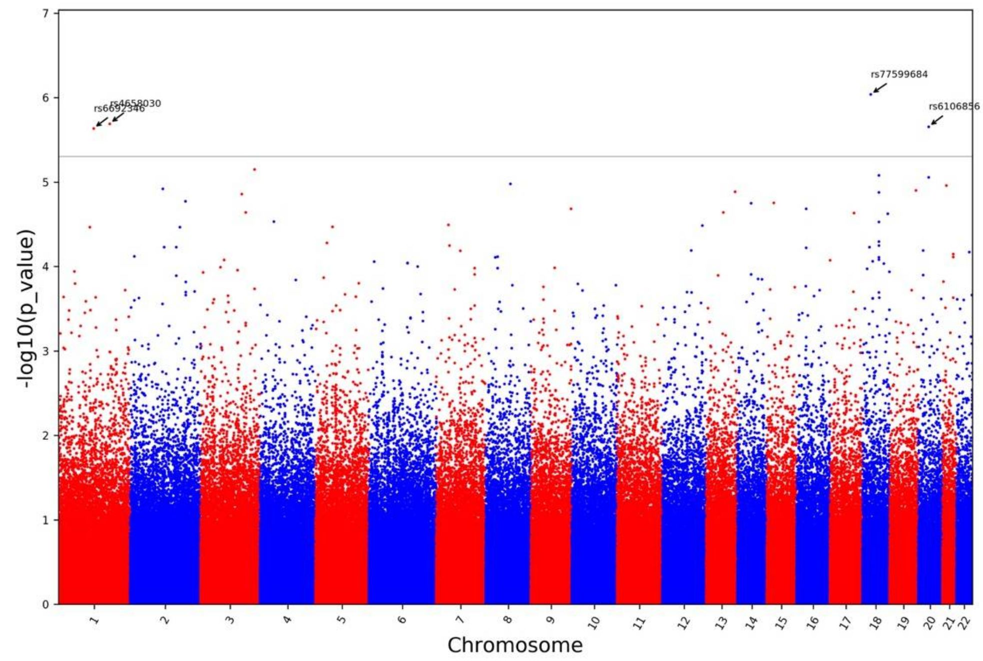

(**e**)

Figure S1. Linear regression *p-values* for each frequency band. Manhattan plot with the log-transformed *p-values* across each chromosome, highlighted by interlaced colors. The grey line marks the custom threshold at 5e-06. Variants that pass the threshold are highlighted by an arrow and the rsID is displayed. (a) Linear regression *p-values* for the δ frequency band.; (b) Linear regression *p-values* for the θfrequency band.; (c) Linear regression *p-values* for the αfrequency band.; (d) Linear regression *p-values* for the β-1 frequency band.; (e) Linear regression *p-values* for the β-2 frequency band.

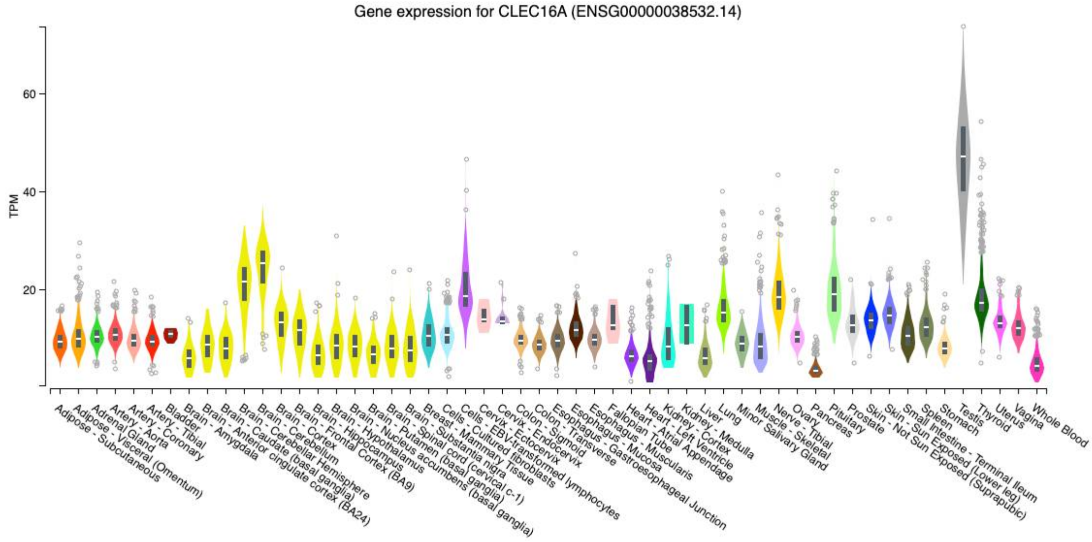

Figure S2. Gene expression for *CLEC16A* in 54 tissues from GTEx v8. Violin plot representing the TPM (transcripts per million) values distribution for each tissue. Every main tissue type is represented with a different color. The dots represent outlying values.

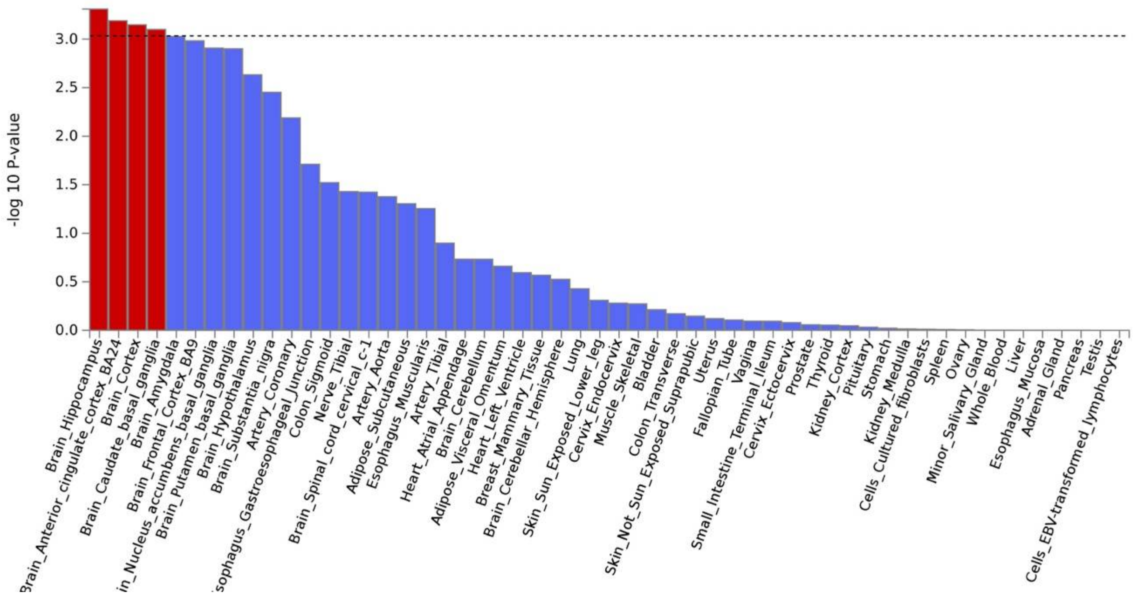

Figure S3. Significantly enriched up-regulated DEGs (differentially expressed genes) for the 15 lead SNPs on GTEx v8 54 tissue types. Log-transformed *p-values* for the enrichment of up-regulated DEGs in each tissue. Significantly enriched tissues are displayed in red (adjusted *p-value* ≤ 0.05), otherwise in blue. The dashed line represents the significance threshold.

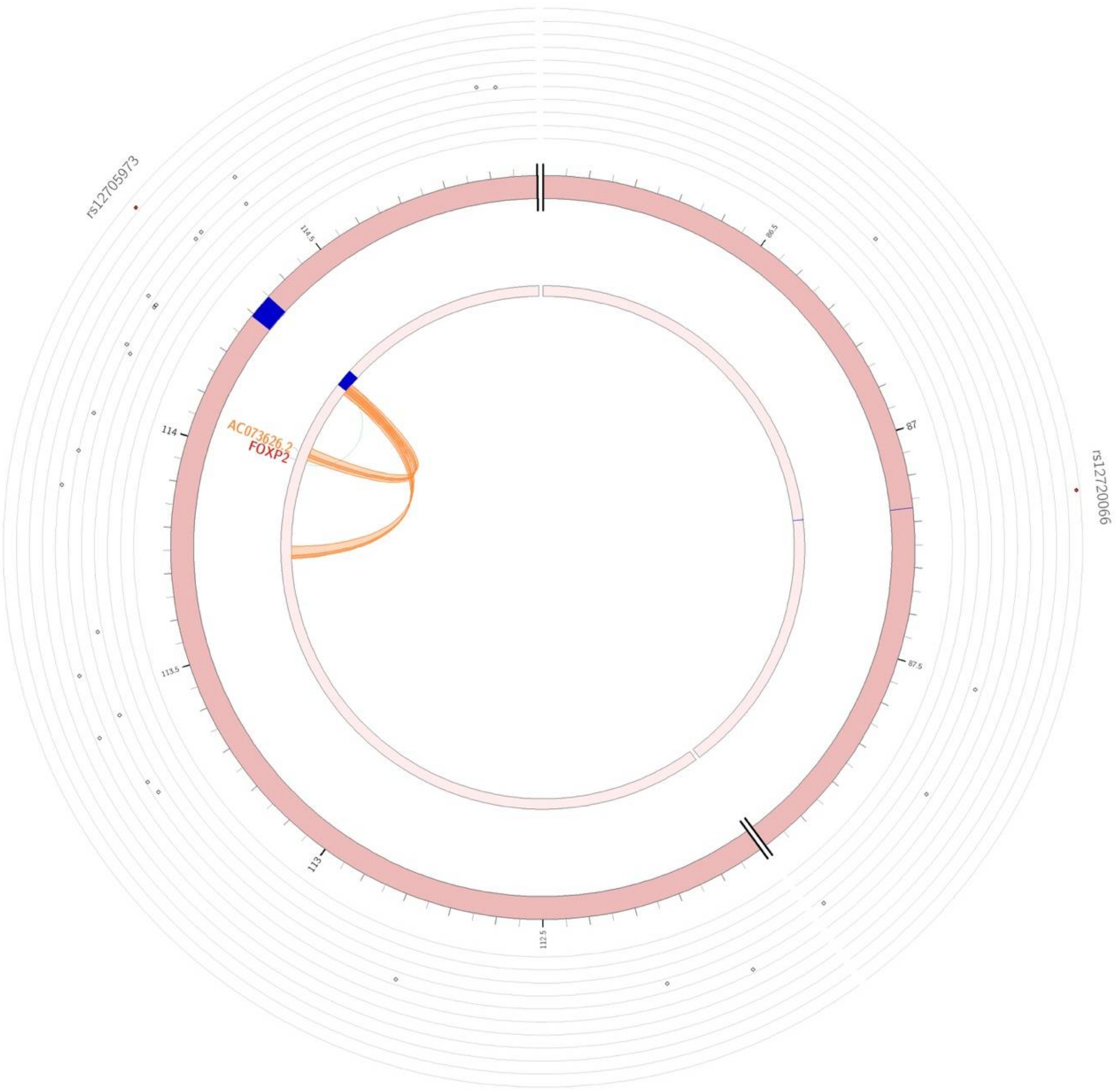

Figure S4. Circos plot for the *rs12705973* genomic associated locus (*FOXP2*). Genomic associated loci are highlighted in blue. Each dot in the outer rings represent a variant. Red dots correspond to variants that pass the defined threshold. If the gene is mapped only by chromatin interactions or only by eQTLs, it is colored orange or green, respectively. When the gene is mapped by both, it is colored red.

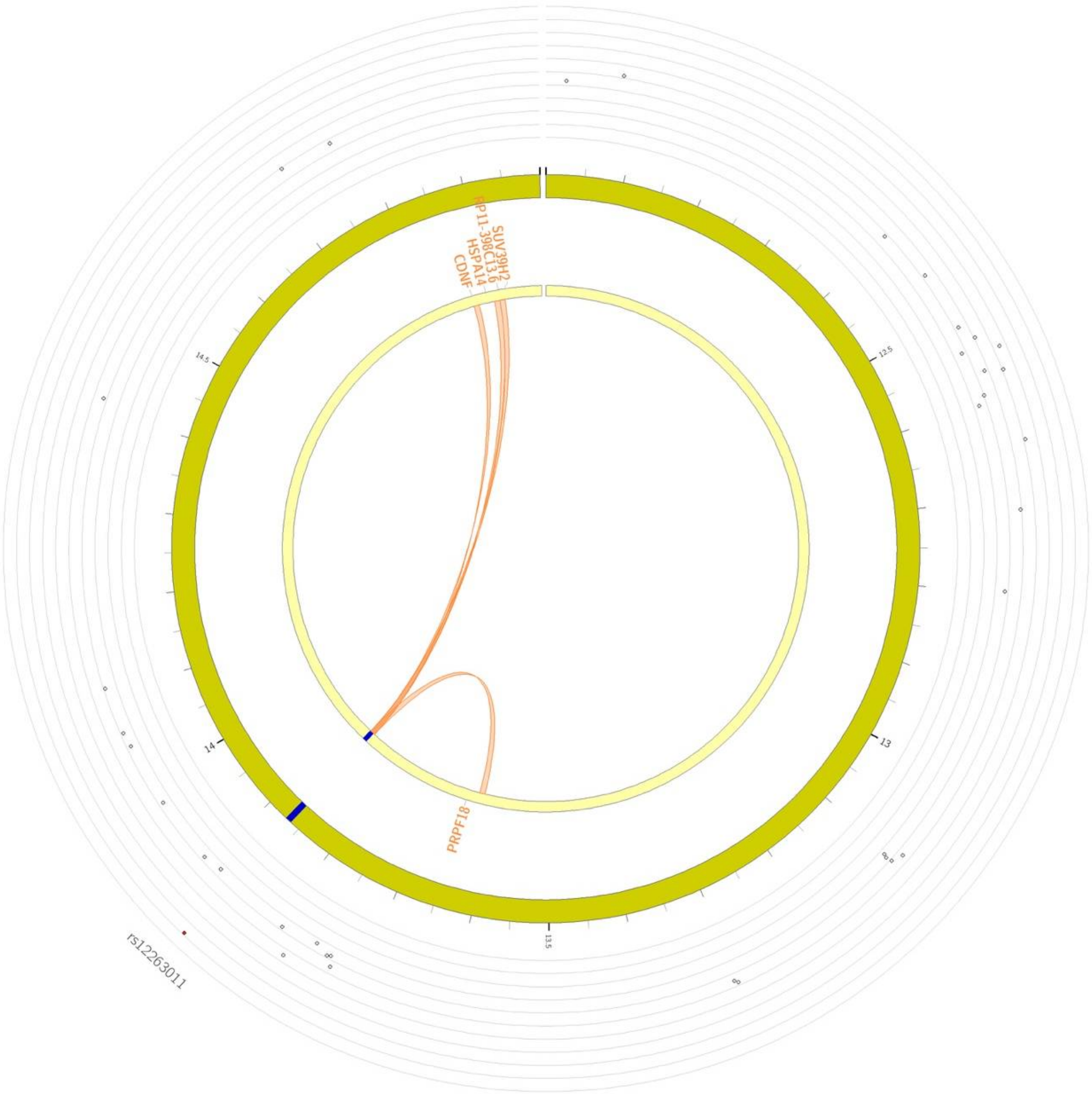

Figure S5. Circos plot for the *rs12705973* genomic associated locus (*FRMD4A*). Genomic associated loci are highlighted in blue. Each dot in the outer rings represent a variant. Red dots correspond to variants that pass the defined threshold. If the gene is mapped only by chromatin interactions or only by eQTLs, it is colored orange or green, respectively. When the gene is mapped by both, it is colored red.

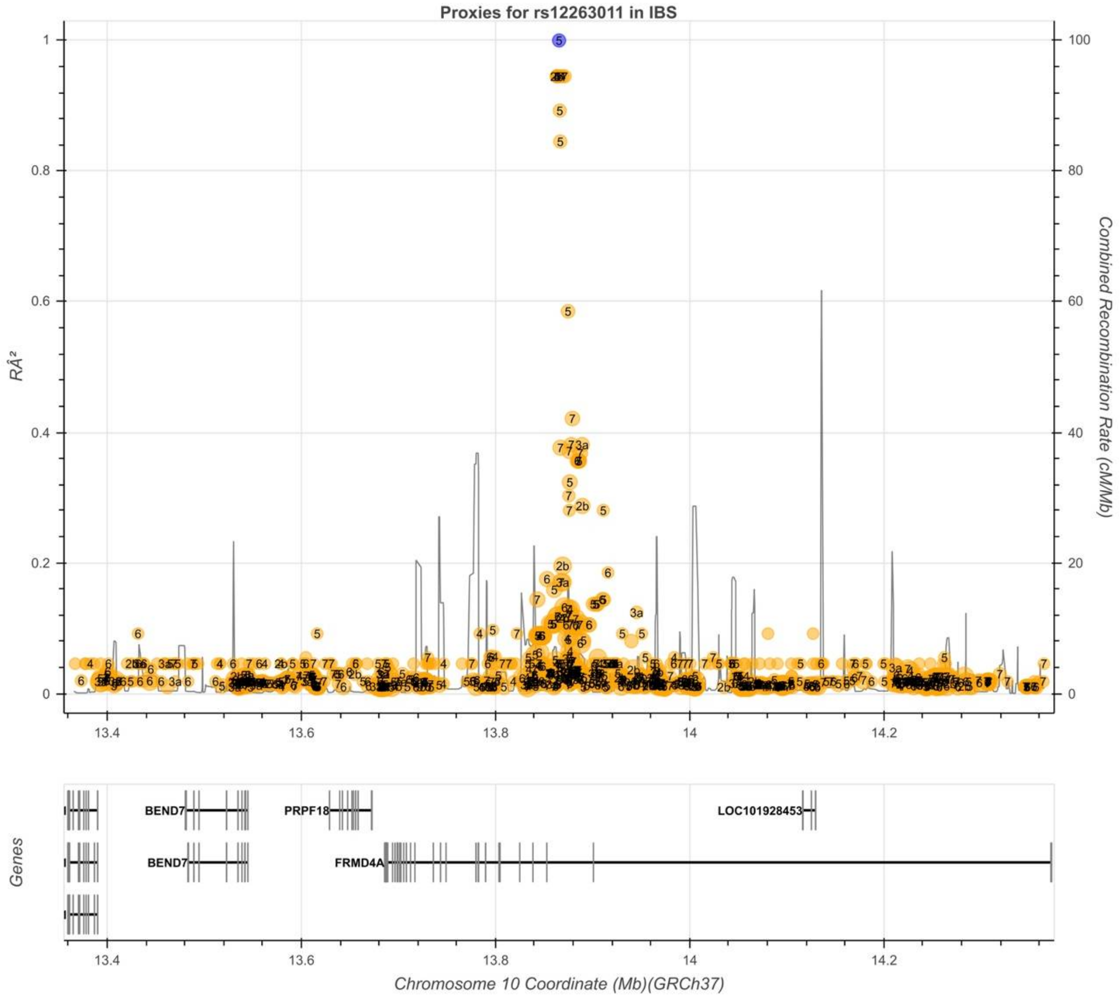

Figure S6. Linkage proxies for the variant *rs12263011*, within *FRMD4A*. Linkage proxies in the 1K Genomes population Iberian in Spain (IBS). The graph displays R^2 (as circles) and combined recombination rate (cM/Mb) (as lines) across chromosome 10 coordinates. The query variant is represented in purple and variants in its vicinity can be classified as non-coding (yellow) or coding (red). The size of the circle is directly proportional to the frequency of the variant. The number inside each circle, if present, indicates the regulatory potential using the scoring scheme of RegulomeDB (http://www.regulomedb.org/). Below the main graph with the R^2 scores the genes are mapped to the chromosome coordinates.

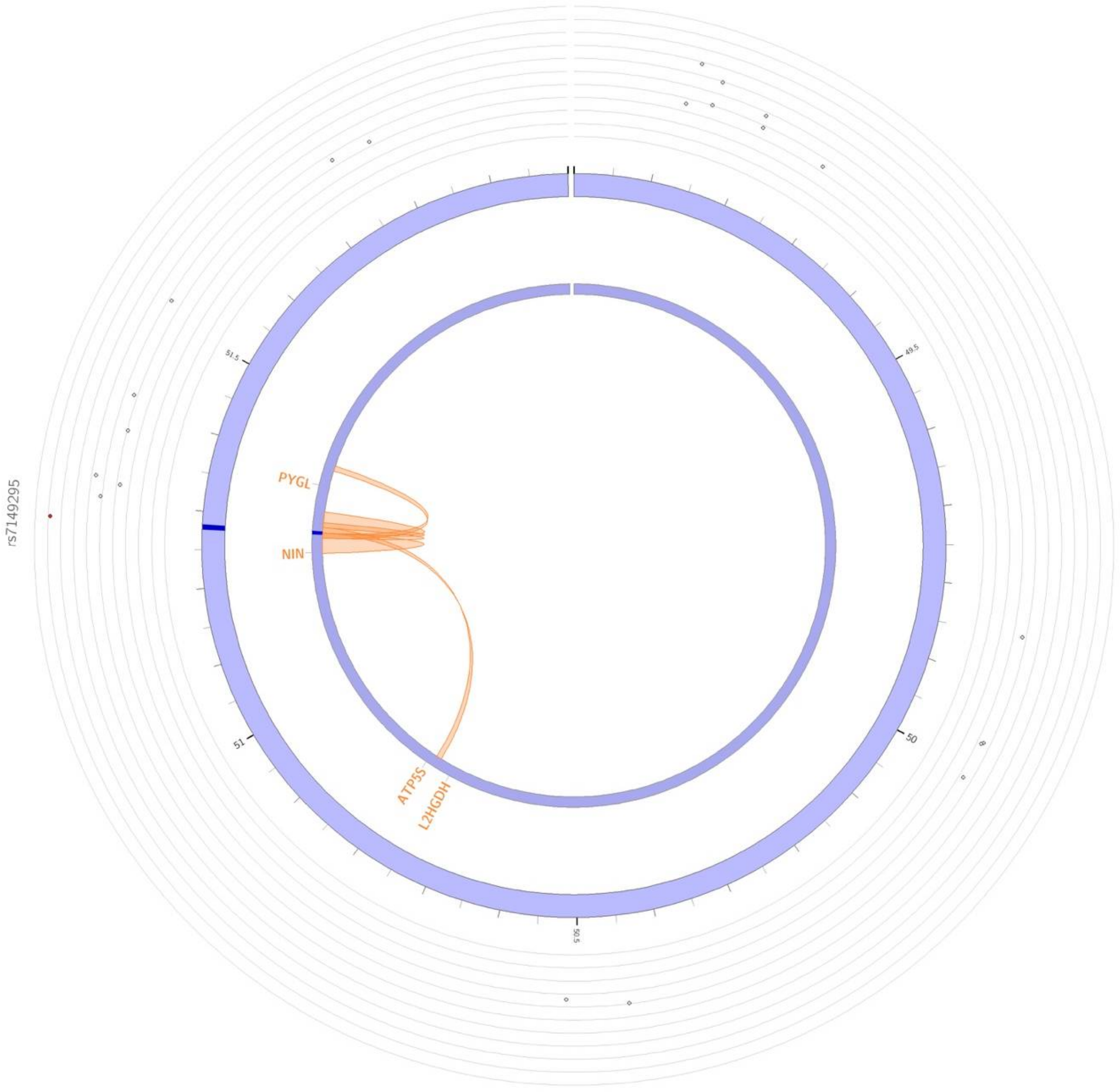

Figure S7. Circos plot for the *rs7149295* genomic associated locus (*L2HGDH*). Genomic associated loci are highlighted in blue. Each dot in the outer rings represent a variant. Red dots correspond to variants that pass the defined threshold. If the gene is mapped only by chromatin interactions or only by eQTLs, it is colored orange or green, respectively. When the gene is mapped by both, it is colored red.

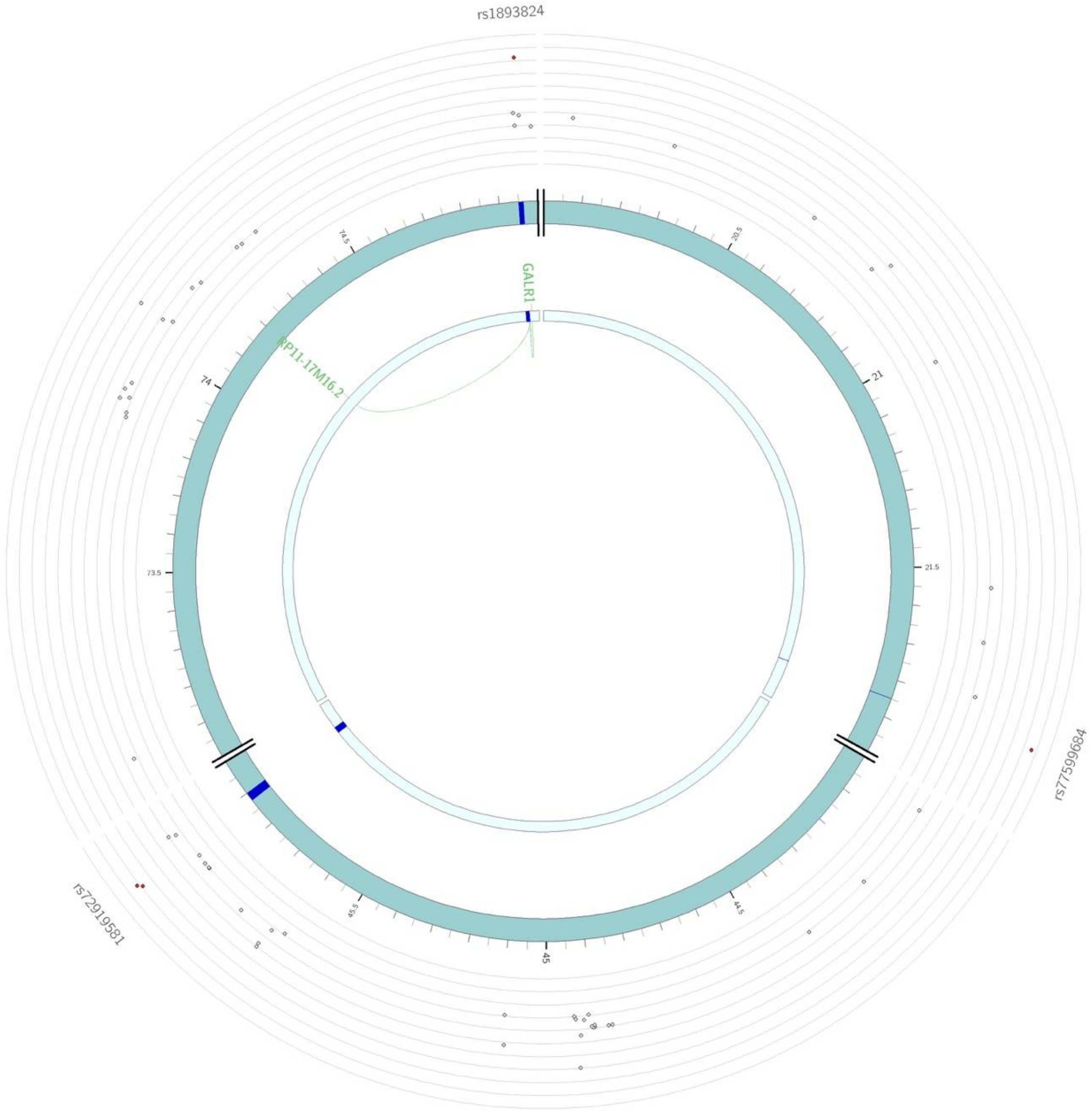

Figure S8. Circos plot for the *rs1893824* genomic associated locus (*GALR1*). Genomic associated loci are highlighted in blue. Each dot in the outer rings represent a variant. Red dots correspond to variants that pass the defined threshold. If the gene is mapped only by chromatin interactions or only by eQTLs, it is colored orange or green, respectively. When the gene is mapped by both, it is colored red.

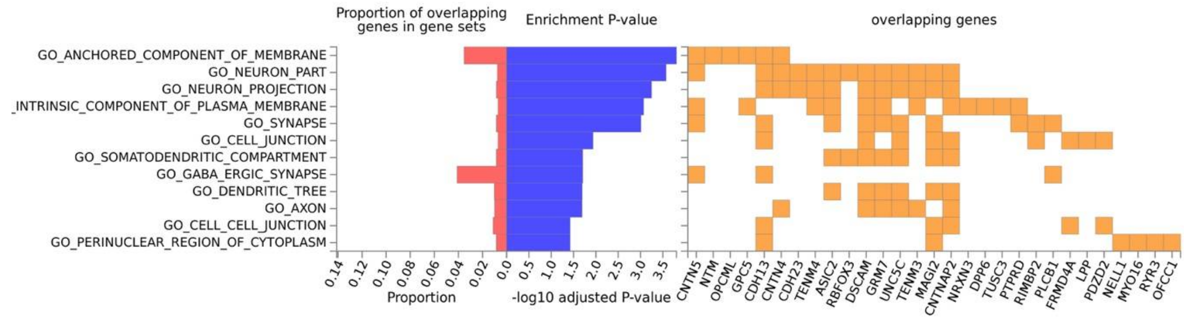

Figure S9. GO (gene-ontology) most significant categories for cellular component. GO terms for cellular components are ordered by enrichment log-transformed *p-value*, displayed in blue. Only statistically significant results are shown. The proportion of overlapping genes for each GO term is displayed in red and for each term the specific genes are identified in yellow.

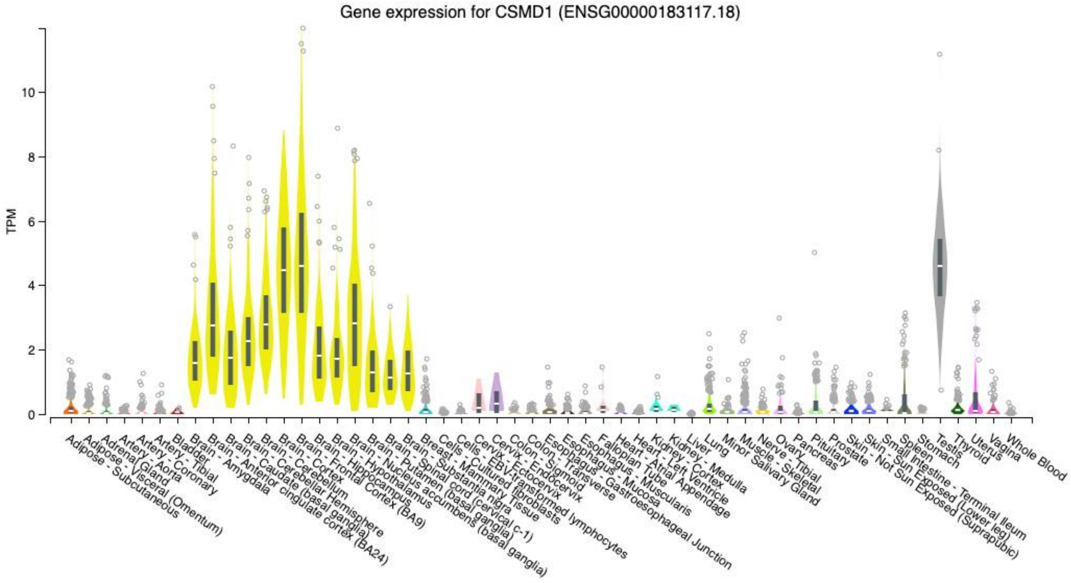

Figure S10. Gene expression for *CSMD1* in 54 tissues from GTEx v8. Violin plot representing the TPM (transcripts per million) values distribution for each tissue. Every main tissue is represented with a different color. The dots represent outlying values.

Table S1. Genescores of genes previously associated with AD. The table displays the number of SNPs mapped to each gene (nSNPs) as well as the computed *p-value* for each gene.

**Gene**	**nSNPs**	**p-value**
CNTNAP2	12.0	8.92×10^{-10}
ALPK2	3.0	4.59×10^{-4}
CLNK	1.0	4.51×10^{-3}
ZCWPW1	1.0	5.95×10^{-3}
ZCWPW1	1.0	5.95×10^{-3}
ADAM10	1.0	7.23×10^{-3}
ECHDC3	1.0	7.26×10^{-3}
ECHDC3	1.0	7.26×10^{-3}
HS3ST1	1.0	7.29×10^{-3}
SLC24A4	1.0	8.57×10^{-3}

Table S2. Genescores of genes previously associated with schizophrenia. The table displays the number of SNPs mapped to each gene (nSNPs) as well as the computed *p-value* for each gene.

**Gene**	**nSNPs**	**p-value**
CNTNAP2	12.0	8.92×10^{-10}
ALPK2	3.0	4.59×10^{-4}
CLNK	1.0	4.51×10^{-3}
ZCWPW1	1.0	5.95×10^{-3}
ZCWPW1	1.0	5.95×10^{-3}
ADAM10	1.0	7.23×10^{-3}
ECHDC3	1.0	7.26×10^{-3}
ECHDC3	1.0	7.26×10^{-3}
HS3ST1	1.0	7.29×10^{-3}
SLC24A4	1.0	8.57×10^{-3}

© 2020 by the authors. Submitted for possible open access publication under the terms and conditions of the Creative Commons Attribution (CC BY) license (http://creativecommons.org/licenses/by/4.0/).
